# Supplementary material for: The CDK12 inhibitor SR-4835 functions as a molecular glue that promotes cyclin K degradation in melanoma
Source: Cell Death Discov. 2023 Dec 16;9:459. doi: 10.1038/s41420-023-01754-x (PMC10725499; doi:10.1038/s41420-023-01754-x)
Supplement: Supplementary file 1 — Supplemental Material [file 41420_2023_1754_MOESM1_ESM.pdf]

Supporting information

The CDK12 inhibitor SR-4835 functions as a molecular glue that promotes cyclin K degradation in melanoma

Thibault Houles,[1,7,*] Jonathan Boucher,[1,7] Geneviève Lavoie,[1] Graham MacLeod,[2] Sichun Lin,[2] Stephane Angers,[2,3,4] Philippe P. Roux[1,4,5,6,*]

[1]Institute for Research in Immunology and Cancer (IRIC), Université de Montréal, Montreal, 2950, Chemin de la Polytechnique, Montréal, QC, H3T 1J4, Canada.

[2]Donnelly Centre for Cellular & Biomolecular Research, Temerty Faculty of Medicine, University of Toronto, Toronto, ON, Canada.

[3]Leslie Dan Faculty of Pharmacy, University of Toronto, Toronto, Canada.

[4]Department of Biochemistry, Temerty Faculty of Medicine, University of Toronto, Toronto, ON, Canada.

[4]Department of Pathology and Cell Biology, Faculty of Medicine, Université de Montréal, Montreal, Quebec, Canada.

[5]Senior author

[6]Lead contact

[7]These authors contributed equally.

[*]Correspondence: philippe.roux@umontreal.ca (P.P.R.) or thibault.houles@igmm.cnrs.fr (T.H.)

Running title: SR-4835 promotes cyclin K degradation

Table of contents

Figure S1. Summary and HPLC spectrum of SR-4835	3
Figure S2. LC-MS analysis of SR-4835	4
Figure S3. Summary and HPLC spectrum of CU-0904	5
Figure S4. LC-MS analysis of CU-0904	6
Figure S5. 1H NMR spectrum of CU-0904	7
Figure S6. 19F NMR spectrum of CU-0904	8
Figure S7. Summary and HPLC spectrum of CU-0905	9
Figure S8. LC-MS analysis of CU-0905	10
Figure S9. 1H NMR spectrum of CU-0905	11
Figure S10. 19F NMR spectrum of CU-0905	12
Figure S11. Summary and HPLC spectrum of CU-0906	13
Figure S12. LC-MS analysis of CU-0906	14
Figure S13. 1H NMR spectrum of CU-0906	15

Chromatogram and Results

Injection Details			
Injection Name:	**BIZ2022-N09-SCH13**	*Run Time (min):*	**16.00**
Vial Number: Injection	**GC2**	*Injection Volume:*	**2.00**
Type:	**Unknown**	*Channel:*	**UV_VIS_1**
CAS NO.:	**2387704-62-1-A-RP-1283**	*Wavelength:*	**214**
Instrument Method:	**10-90A**	*Bandwidth:*	**4**
Processing Method:	**3D Quantitative_1**	*Dilution Factor:*	**1.0000**
Injection Date/Time:	**12/Nov/22 11:51 WangShanLi**	seq.update_operator	**wsl**
seq.creation_operator	**1.0000**	instMeth.creation_operator	**ly**
injection.dilution_factor	**22/Jan/2021 15:28**	rdf.update_operator	**wsl**
instMeth.creation_time			
chm.rawpath	**chrom://hy583/HPLC-63/202211/20221112.seq/762.smp/UV_VIS_1.channel/UV_VIS_1.chm**		

Chromatogram

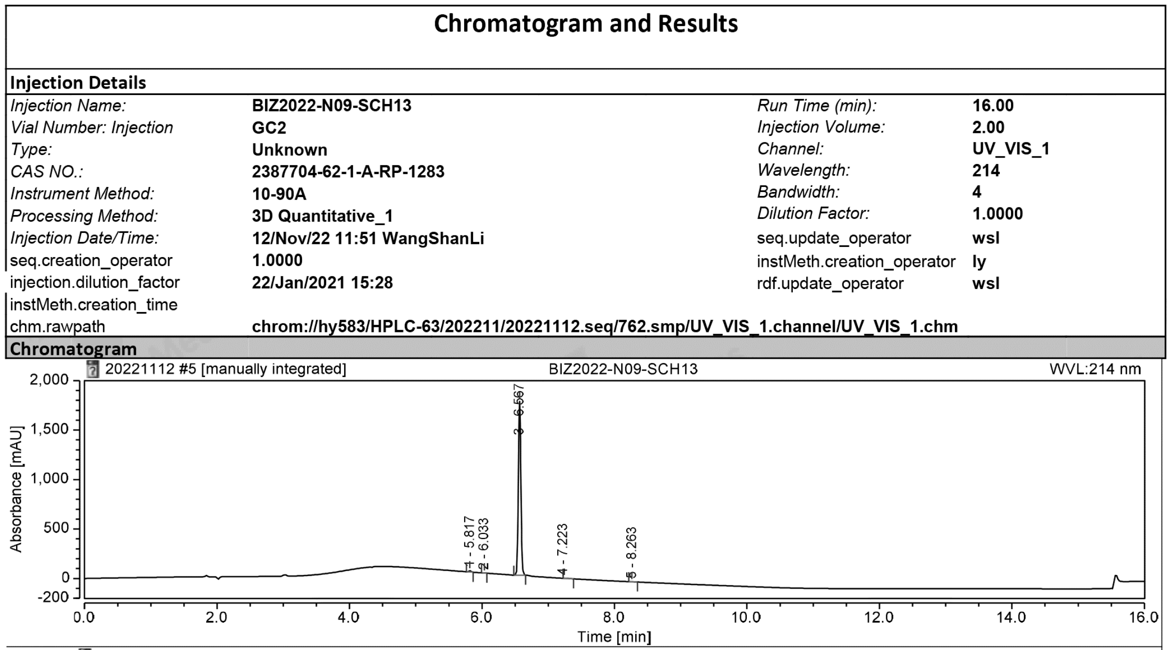

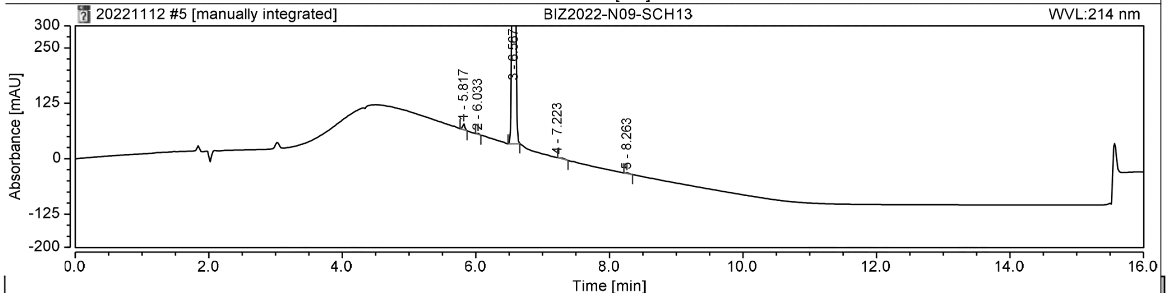

No.	Peak Name	Retention Time min	Area mAU*s	Height mAU	Relative Area %	Resolution (USP)	Match	S/N
1		5.817	30.7552	13.354	0.6641	n.a.	1000	19.7
2		6.033	5.1581	2.408	0.1114	3.6	996	5.1
3		6.567	4579.3187	1754.554	98.8853	8.5	1000	535.7
4		7.223	8.9262	0.000	0.1928	n.a.	1000	n.a.
5		8.263	6.7816	1.930	0.1464	n.a.	991	3.7
**Total:**			**4630.9399**	**1772.246**	**100.0000**		**4986**	

Figure S1. Summary and HPLC spectrum of SR-4835

Match Plot

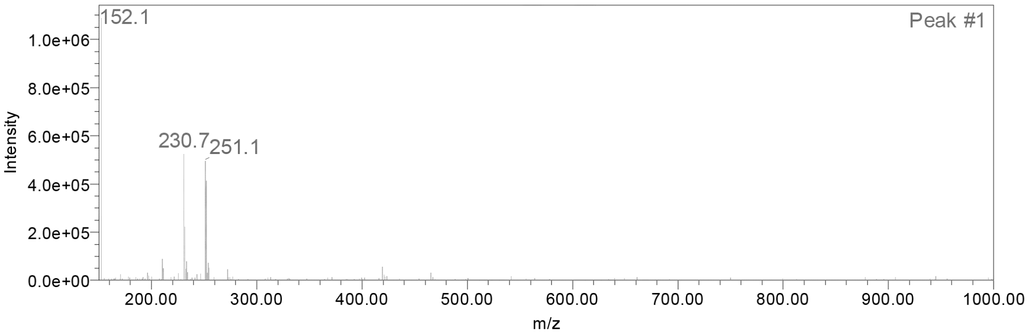

Retention Time 0.723 Channel Name MS TIC

Peak Results
Channel Name: MS TIC

	RT	Width (sec)	Area	Height	% Area	Base Peak (Combined) (m/z)	Channel Name
1	0.723	2.611	6081839	3111997	2.42	150.93	MS TIC
2	0.854	5.803	198213859	90662977	78.90	270.65	MS TIC
3	0.992	4.933	46914129	17460069	18.68	152.02	MS TIC

Match Plot

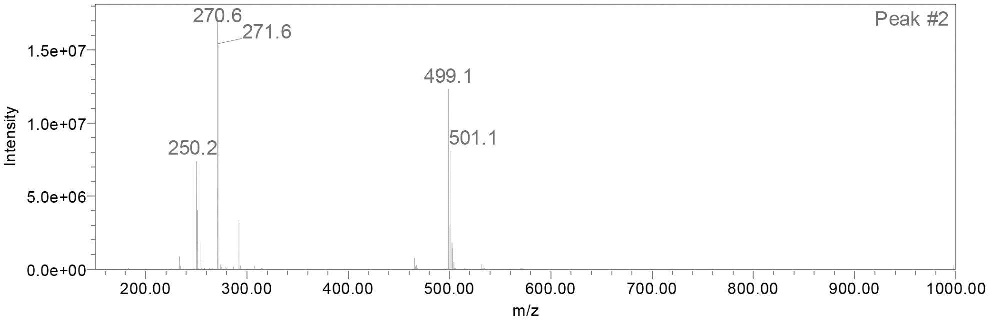

Retention Time 0.854 Channel Name MS TIC

Match Plot

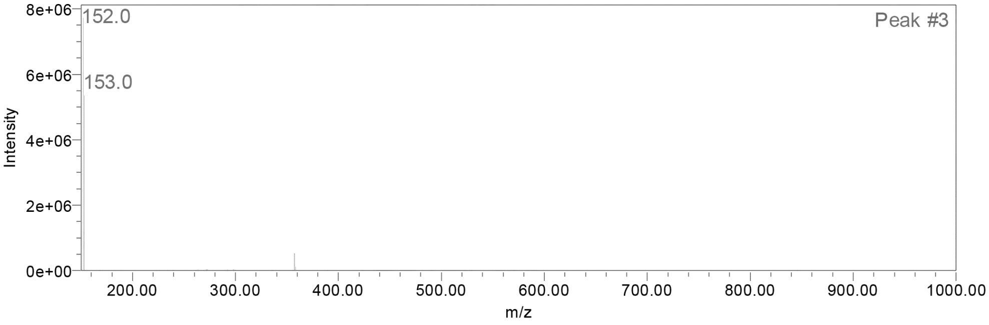

Retention Time 0.992 Channel Name MS TIC

Figure S2. LC-MS analysis of SR-4835

Description		
Name	9*H*-Purin-6-amine, *N*-[(5,6-difluoro-1*H*-benzimidazol-2-yl)methyl]-9-(1-methyl-1*H*-pyrazol-4-yl)-2-(4-morpholinyl)-	
OmegaChem #	CU-0904	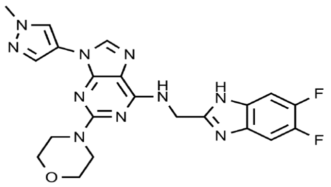
Lot #	01MD519-329-02	
CAS #	2387704-56-3	
Chemical Formula	$C_{21}H_{20}F_2N_{10}O$	
Molecular Weight	466.45	
Campaign	C0737-0904-01-21	

Analysis		
Tests	Specifications	Results
Appearance	White to Pale Yellow Solid	Off-white solid
Chemical Purity	95% minimum (HPLC)	99.8 % (average λ = 215 and λ = 254 nm)
Mass Spectroscopy	Must comply to Structure	$[M+H]^+ = 467.2$
^1H NMR	Must comply to Structure	Comforms (DMSO-d_6)
^{19}F NMR	Must comply to Structure	Comforms (DMSO-d_6)

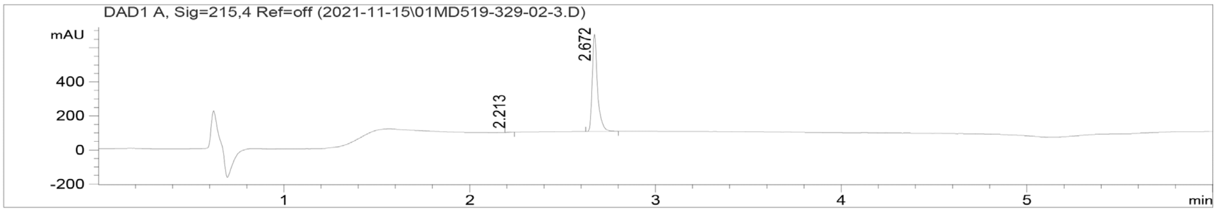

#	Meas. Ret. Ti	Height	Height %	Area	Area %
1	2.213	2.708	0.473	4.025	0.356
2	2.672	569.482	99.527	1126.317	99.644

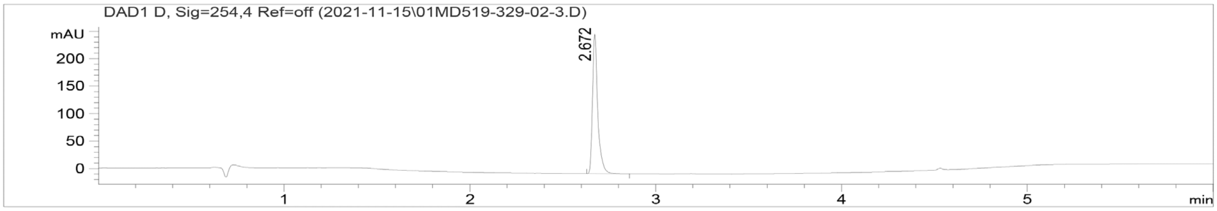

#	Meas. Ret. Ti	Height	Height %	Area	Area %
1	2.672	253.886	100.000	501.344	100.000

Figure S3. Summary and HPLC spectrum of CU-0904

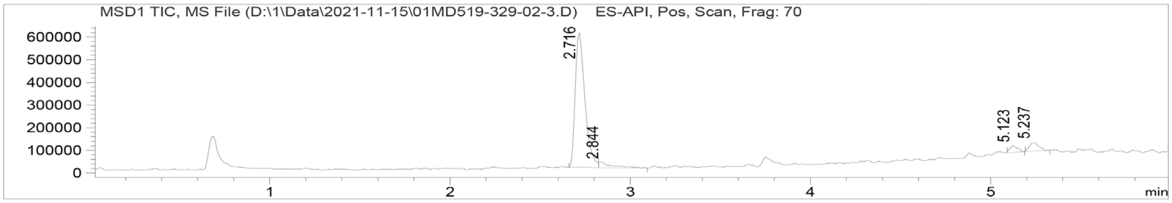

#	Meas. Ret. Ti	Height	Height %	Area	Area %
1	2.716	596249.063	86.359	2224277.250	86.364
2	2.844	24802.652	3.592	129612.992	5.033
3	5.123	30769.904	4.457	89153.016	3.462
4	5.237	38605.496	5.592	132416.359	5.141

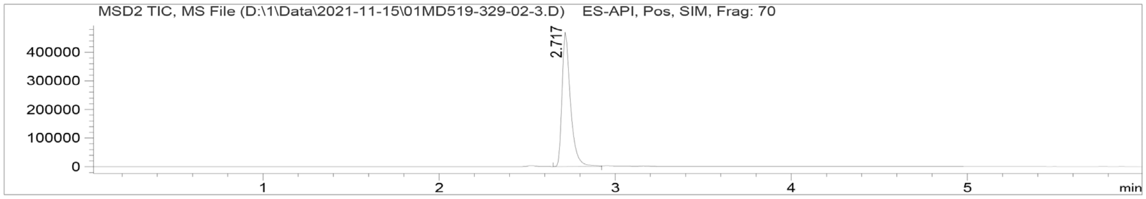

#	Meas. Ret. Ti	Height	Height %	Area	Area %
1	2.717	472104.781	100.000	1567202.875	100.000

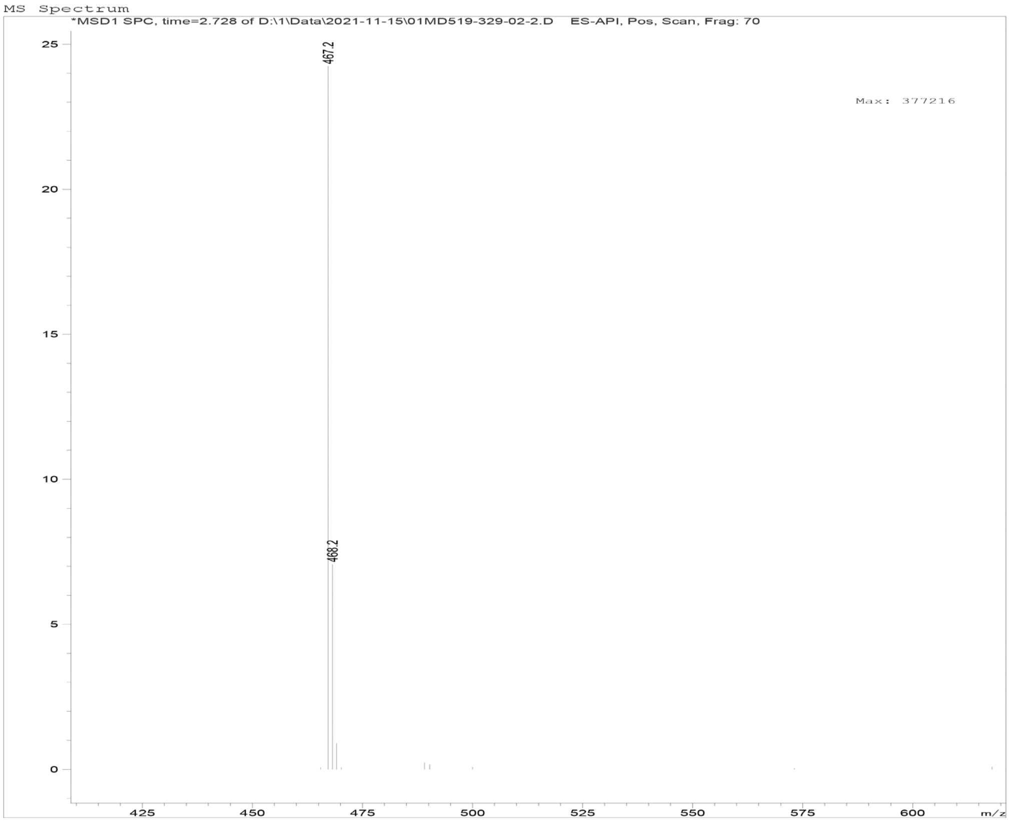

Figure S4. LC-MS analysis of CU-0904

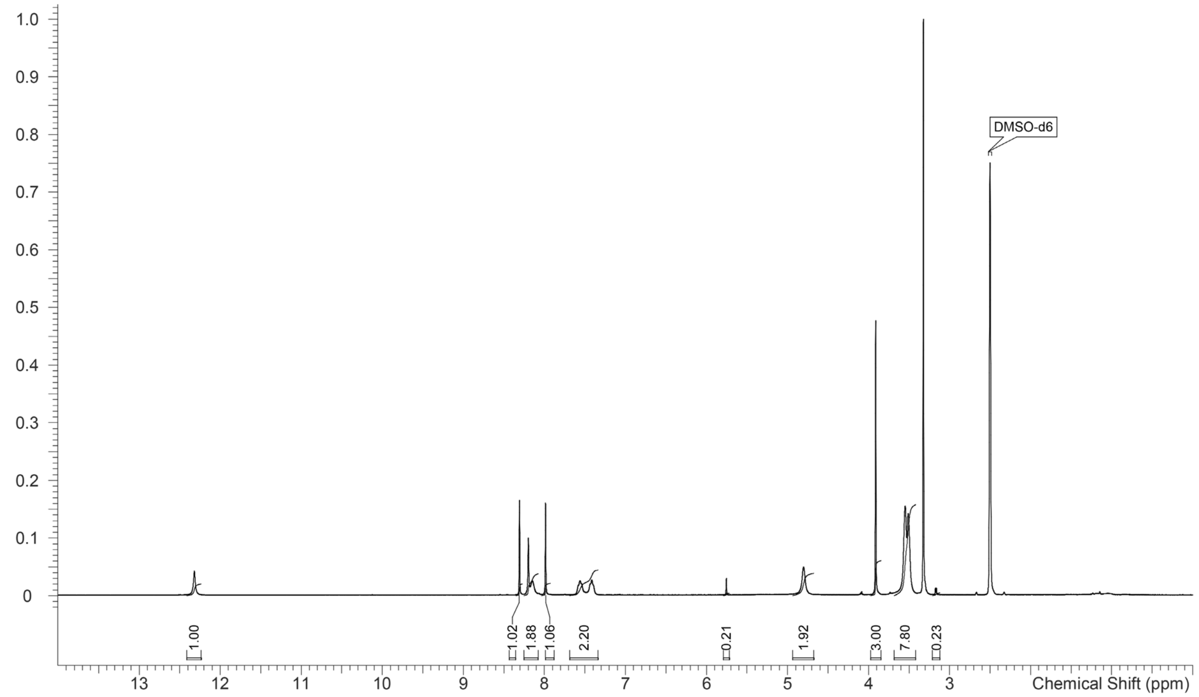

^{1}H NMR (400 MHz, DMSO-d_6) δ ppm 3.42 - 3.68 (m, 8 H), 3.91 (s, 3 H), 4.80 (br s, 2 H), 7.34 - 7.69 (m, 2 H), 7.99 (s, 1 H), 8.07 - 8.25 (m, 2 H), 8.31 (s, 1 H), 12.32 (br s, 1 H).

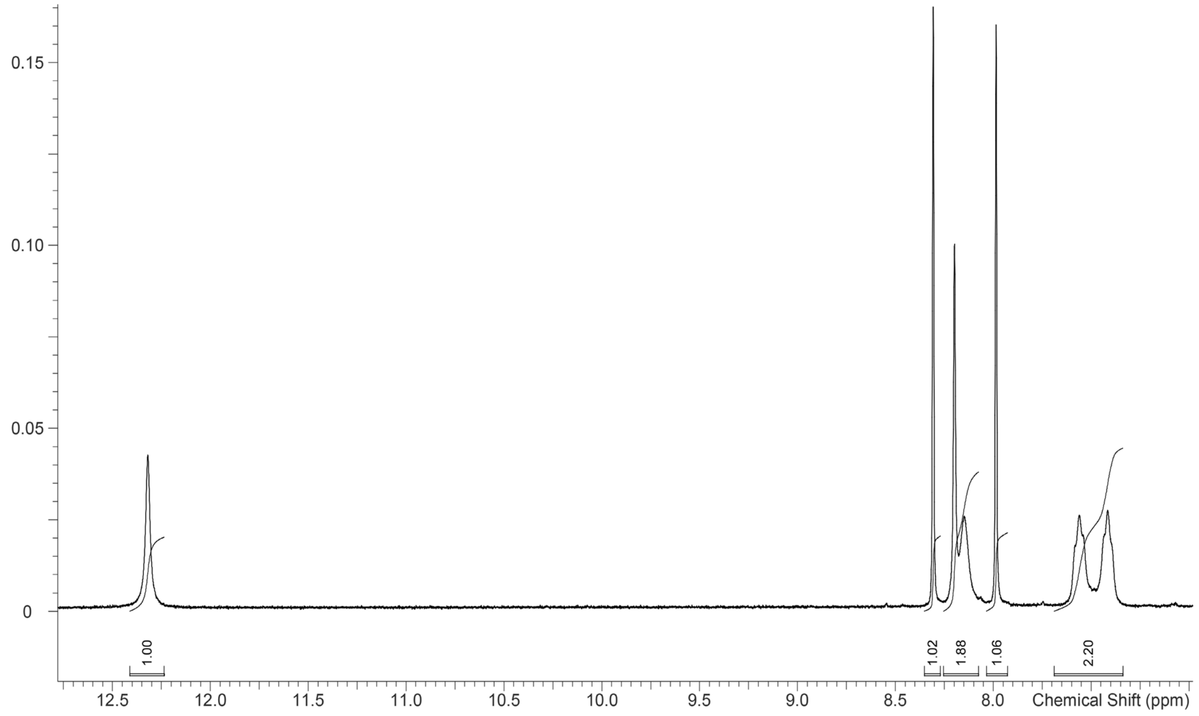

^{1}H NMR (400 MHz, DMSO-d_6) δ ppm 3.42 - 3.68 (m, 8 H), 3.91 (s, 3 H), 4.80 (br s, 2 H), 7.34 - 7.69 (m, 2 H), 7.99 (s, 1 H), 8.07 - 8.25 (m, 2 H), 8.31 (s, 1 H), 12.32 (br s, 1 H).

Figure S5. 1H NMR spectrum of CU-0904

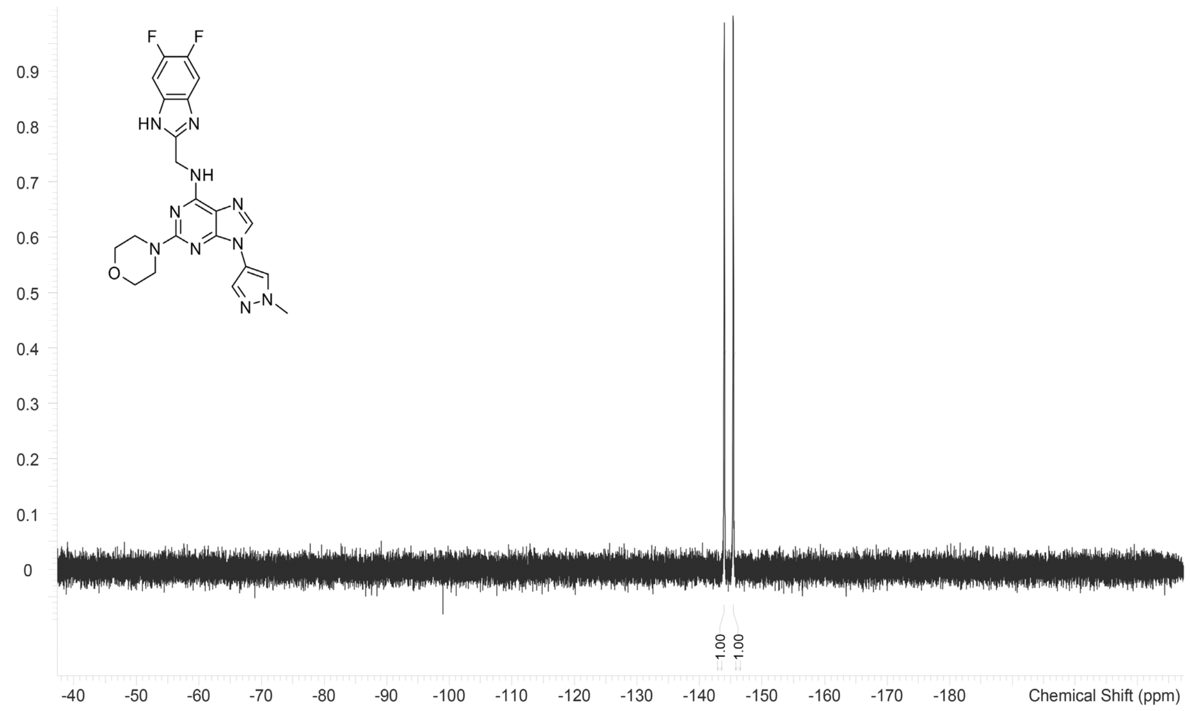

^{19}F NMR (377 MHz, DMSO-d_6) δ ppm -146.38 (br. s, 1 F), -144.97 (br. s, 1 F).

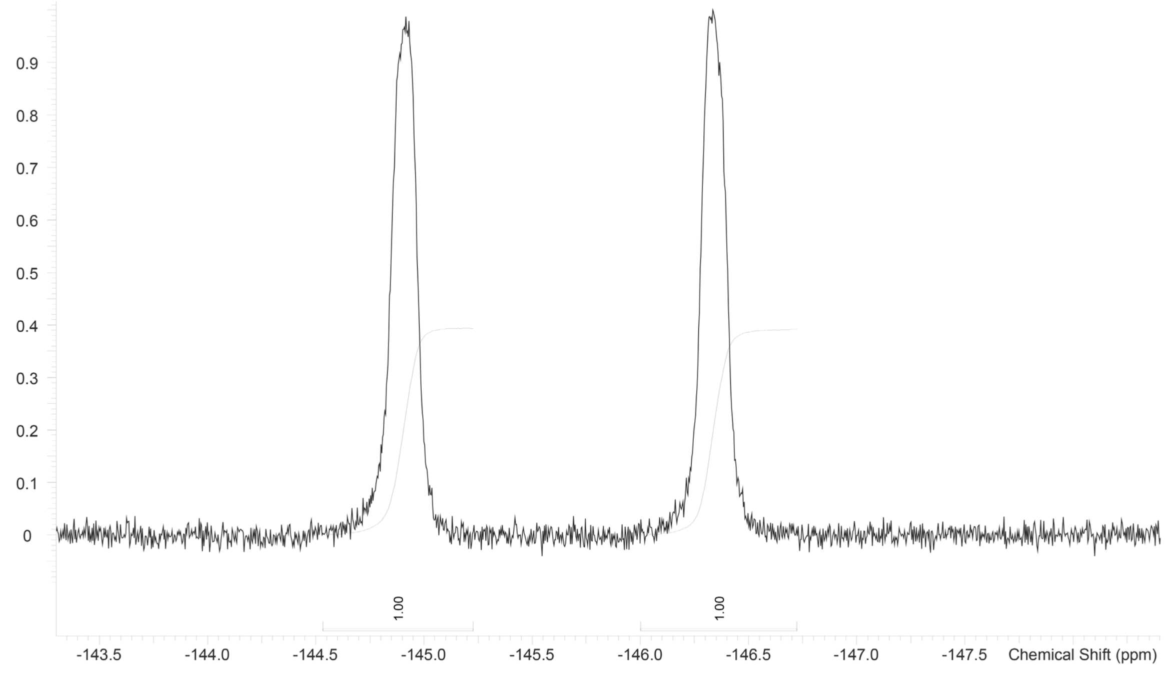

^{19}F NMR (377 MHz, DMSO-d_6) δ ppm -146.38 (br. s, 1 F), -144.97 (br. s, 1 F).

Figure S6. 19F NMR spectrum of CU-0904

Description

Description		
Name	9*H*-Purin-6-amine, *N*-[(6,7-difluoro-1*H*-benzimidazol-2-yl)methyl]-9-(1-methyl-1*H*-pyrazol-4-yl)-2-(4-morpholinyl)-	
OmegaChem #	CU-0905	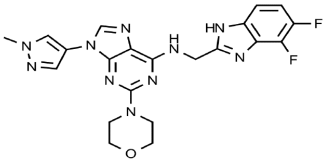
Lot #	01MD519-328-03	
CAS #	2387704-54-1	
Chemical Formula	$C_{21}H_{20}F_2N_{10}O$	
Molecular Weight	466.45	
Campaign	C0738-0905-01-21	

Analysis

Tests	Specifications	Results
Appearance	White to Pale Yellow Solid	Off-white solid
Chemical Purity	95% minimum (HPLC)	97.8% (HPLC, average λ = 215 and 254 nm)
Mass Spectroscopy	Must comply to Structure	$[M+H]^+$ = 467.2
^{1}H NMR	Must comply to Structure	Conforms (CD_3OD)
^{19}F NMR	Must comply to Structure	Conforms (CD_3OD)

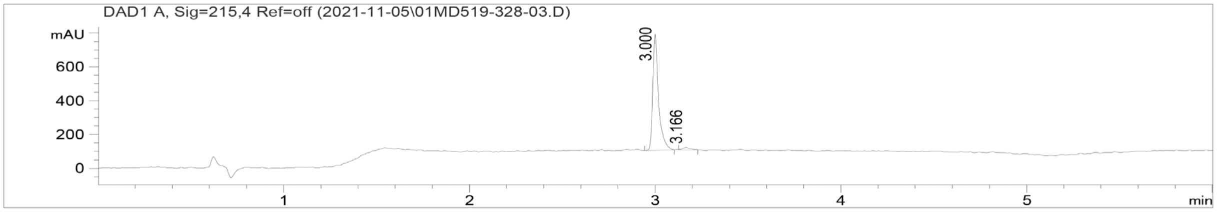

#	Meas. Ret. Ti	Height	Height %	Area	Area %
1	3.000	685.794	98.059	1483.833	97.456
2	3.166	13.575	1.941	38.730	2.544

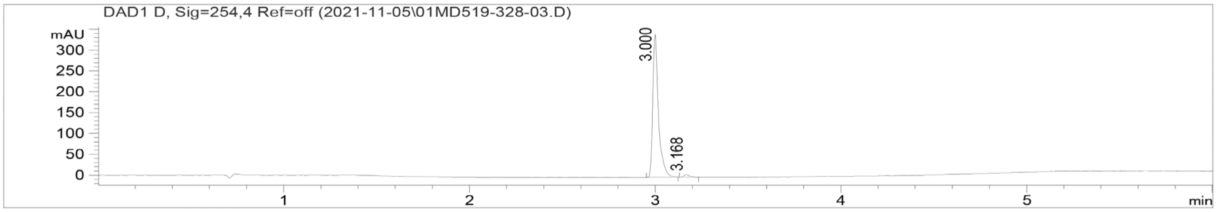

#	Meas. Ret. Ti	Height	Height %	Area	Area %
1	3.000	343.825	98.270	723.396	98.216
2	3.168	6.053	1.730	13.141	1.784

Figure S7. Summary and HPLC spectrum of CU-0905

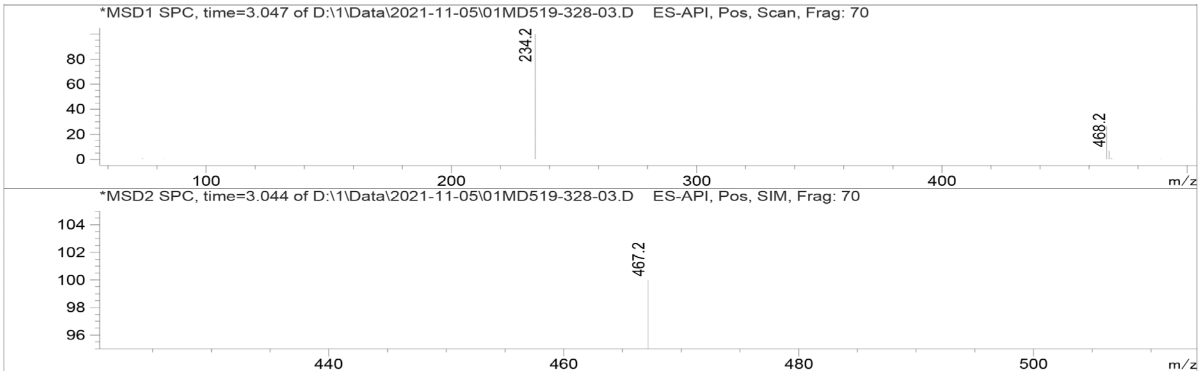

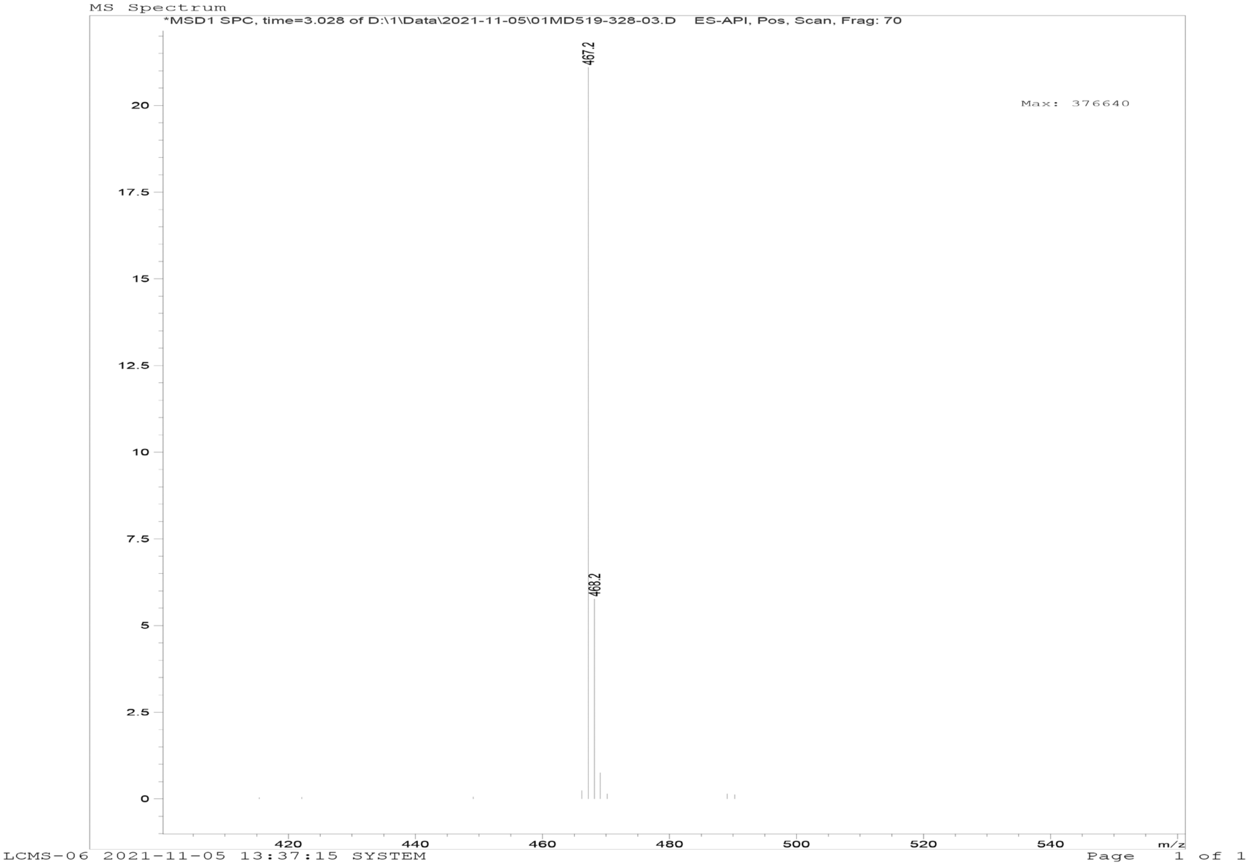

Figure S8. LC-MS analysis of CU-0905

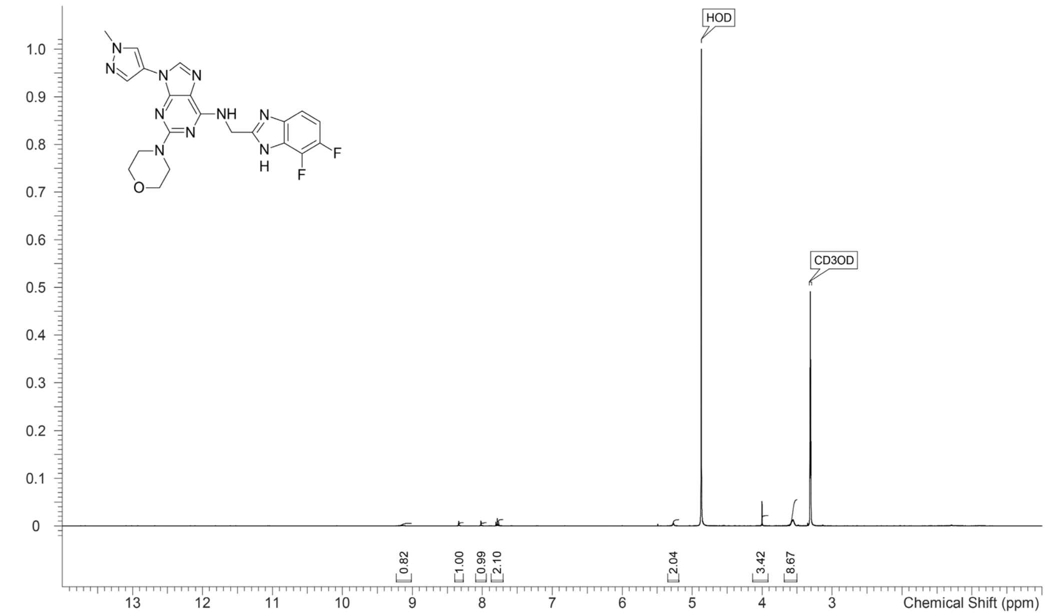

^{1}H NMR (400 MHz, CD_3OD) δ ppm 3.56 (br d, *J* = 5.4 Hz, 9 H), 4.00 (s, 3 H), 5.27 (br s, 2 H), 7.79 (t, *J* = 8.1 Hz, 2 H), 8.02 (s, 1 H), 8.34 (s, 1 H), 9.01 - 9.23 (m, 1 H).

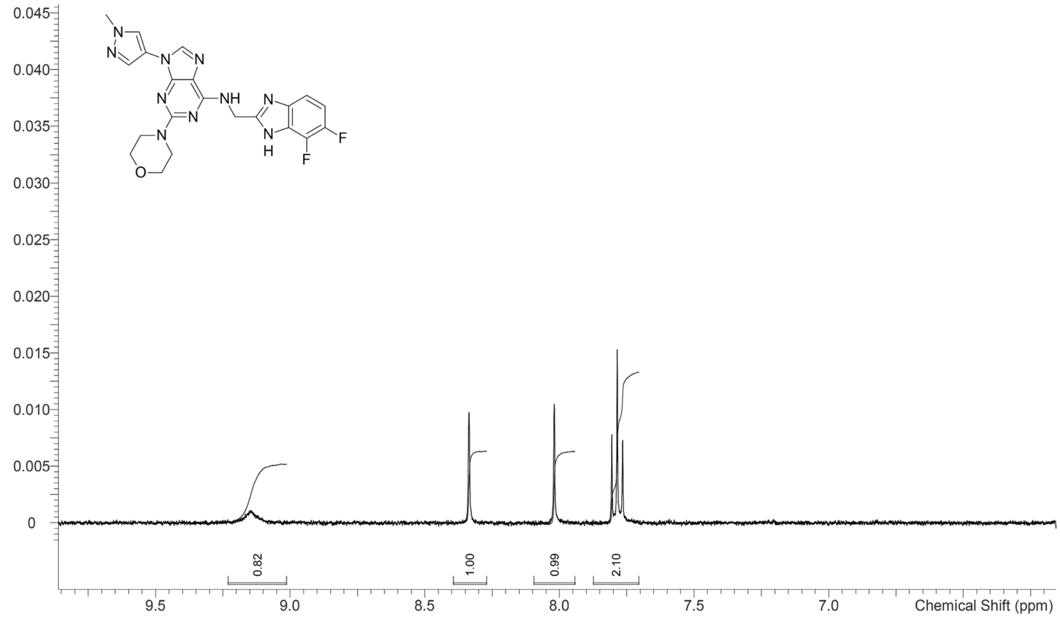

^{1}H NMR (400 MHz, CD_3OD) δ ppm 3.56 (br d, *J* = 5.4 Hz, 9 H), 4.00 (s, 3 H), 5.27 (br s, 2 H), 7.79 (t, *J* = 8.1 Hz, 2 H), 8.02 (s, 1 H), 8.34 (s, 1 H), 9.01 - 9.23 (m, 1 H).

Figure S9. 1H NMR spectrum of CU-0905

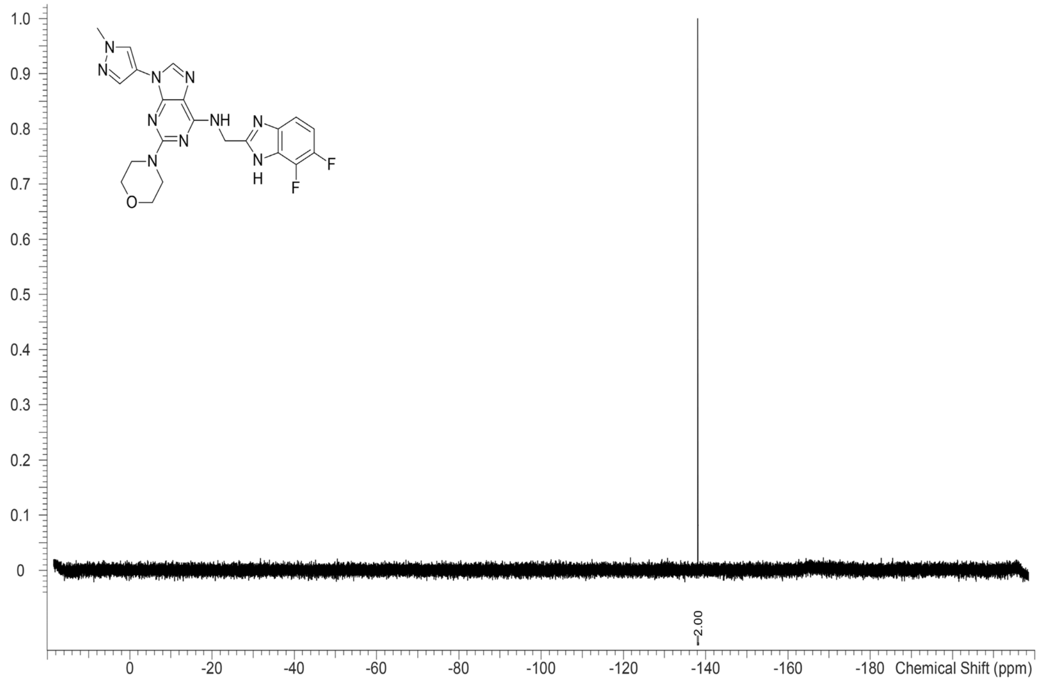

^{19}F NMR (377 MHz, CD_3OD) δ ppm -138.09 (s, 2 F).

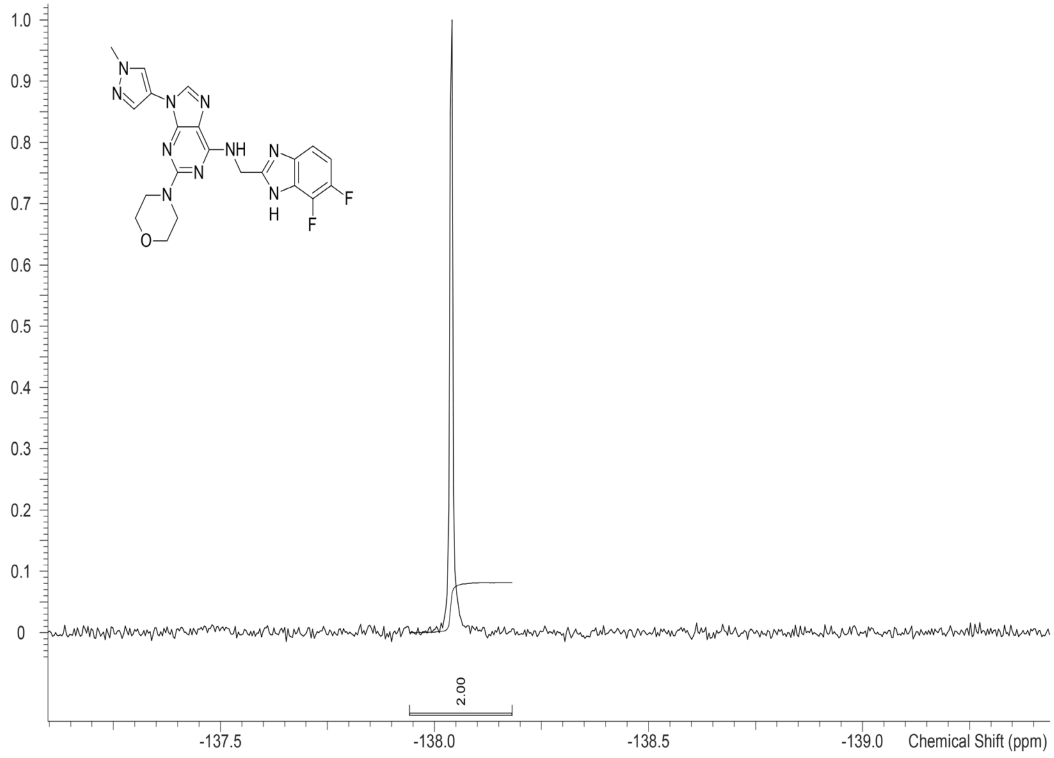

^{19}F NMR (377 MHz, CD_3OD) δ ppm -138.09 (s, 2 F).

Figure S10. 19F NMR spectrum of CU-0905

Description

Name	Composé 3	
OmegaChem #	CU-0906	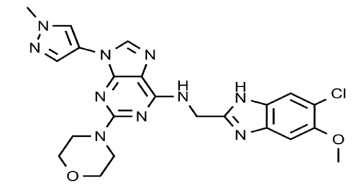
Lot #	01MD519-332-04	
CAS #	N/A	
Chemical Formula	$C_{22}H_{23}ClN_{10}O_2$	
Molecular Weight	494.94	
Campaign	C0739-0906-01-21	

Analysis

Tests	Specifications	Results
Appearance	White to pale-yellow solid	Pale-yellow solid
Chemical Purity	95% minimum (HPLC)	98.4 % (HPLC, average λ = 215 and 254 nm)
Mass Spectroscopy	Must comply to Structure	$[M+H]^+$ = 495.1
^{1}H NMR	Must comply to Structure	Conforms (Methanol-d_4)

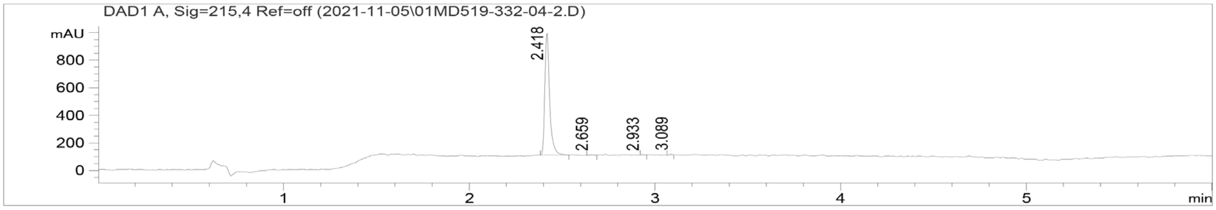

#	Meas. Ret. Ti	Height	Height %	Area	Area %
1	2.418	887.433	98.451	1594.864	98.675
2	2.659	6.537	0.725	13.117	0.812
3	2.933	3.392	0.376	3.997	0.247
4	3.089	4.037	0.448	4.304	0.266

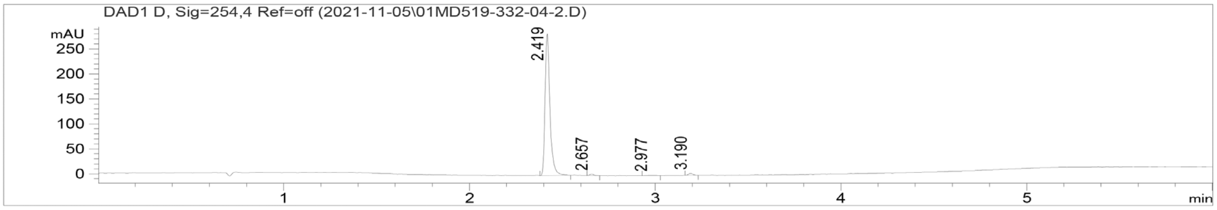

#	Meas. Ret. Ti	Height	Height %	Area	Area %
1	2.419	284.337	97.539	518.246	97.405
2	2.657	2.654	0.911	4.175	0.785
3	2.977	1.005	0.345	3.487	0.655
4	3.190	3.515	1.206	6.142	1.154

Figure S11. Summary and HPLC spectrum of CU-0906

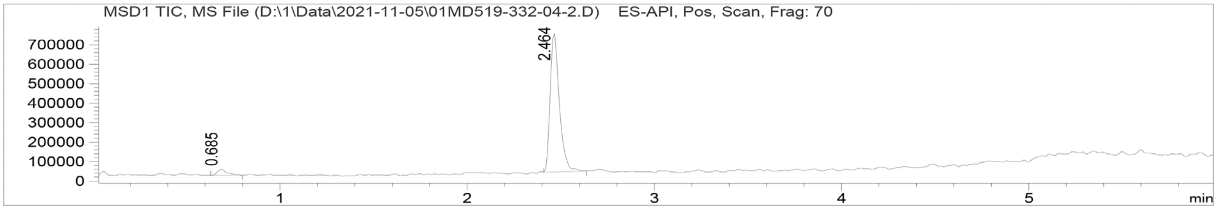

#	Meas. Ret. Ti	Height	Height %	Area	Area %
1	0.685	30547.008	4.083	118031.805	4.614
2	2.464	717573.813	95.917	2440034.750	95.386

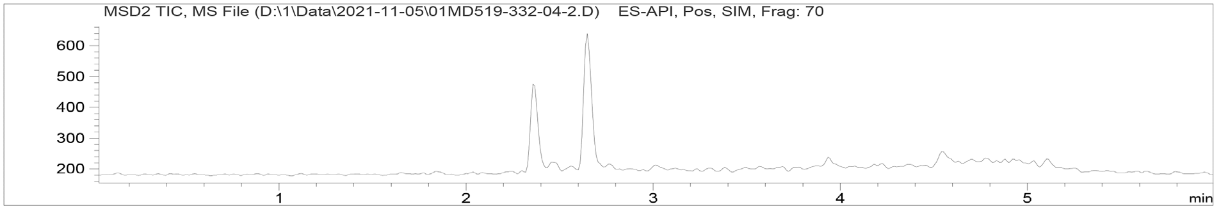

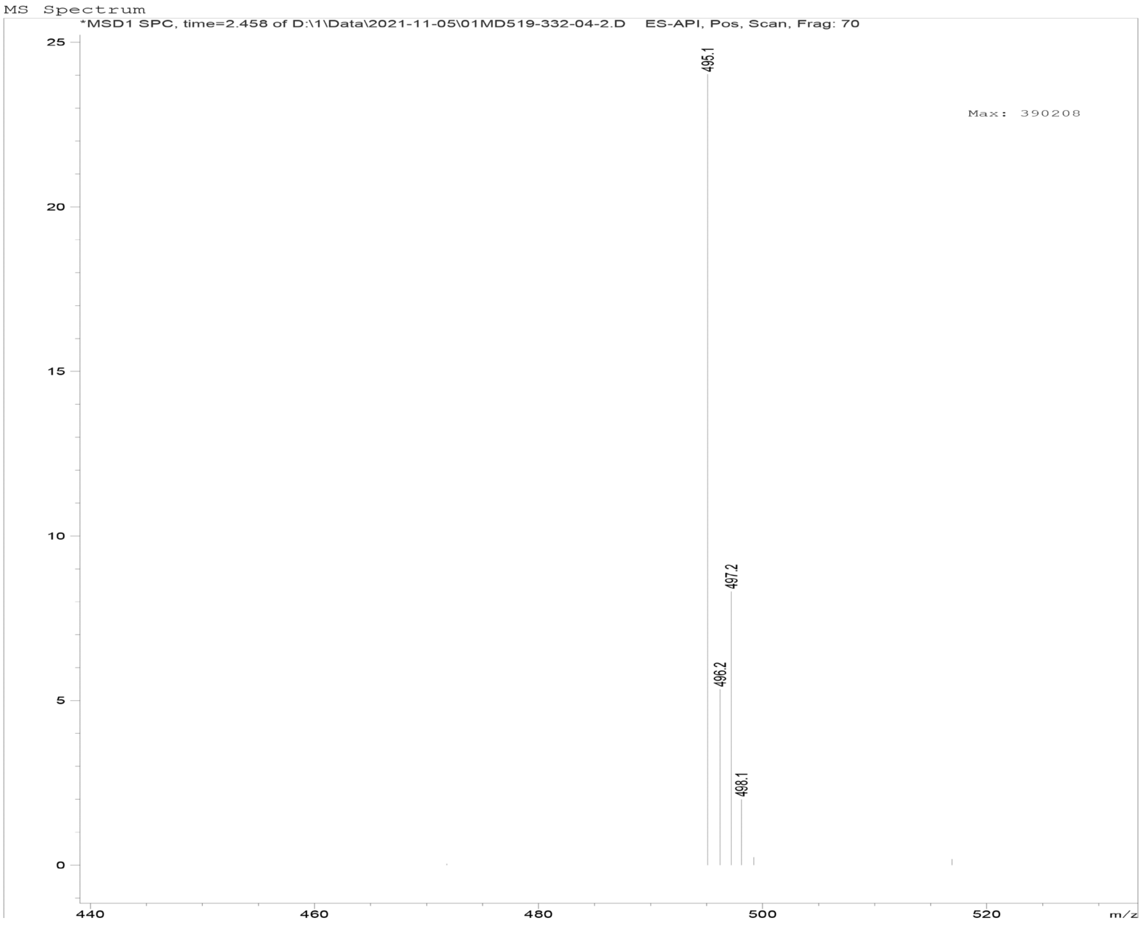

Figure S12. LC-MS analysis of CU-0906

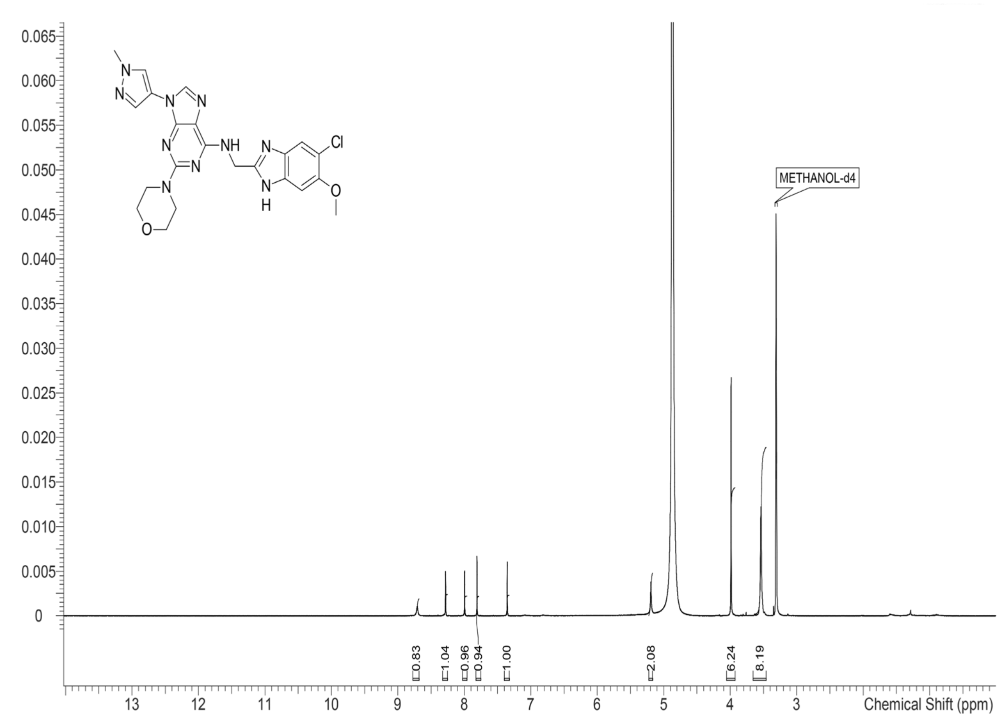

^{1}H NMR (400 MHz, CD_3OD) δ ppm 3.54 (br s, 8 H), 3.99 (s, 6 H), 5.19 (br s, 2 H), 7.36 (s, 1 H), 7.81 (s, 1 H), 8.00 (s, 1 H), 8.28 (s, 1 H), 8.71 (br s, 1 H).

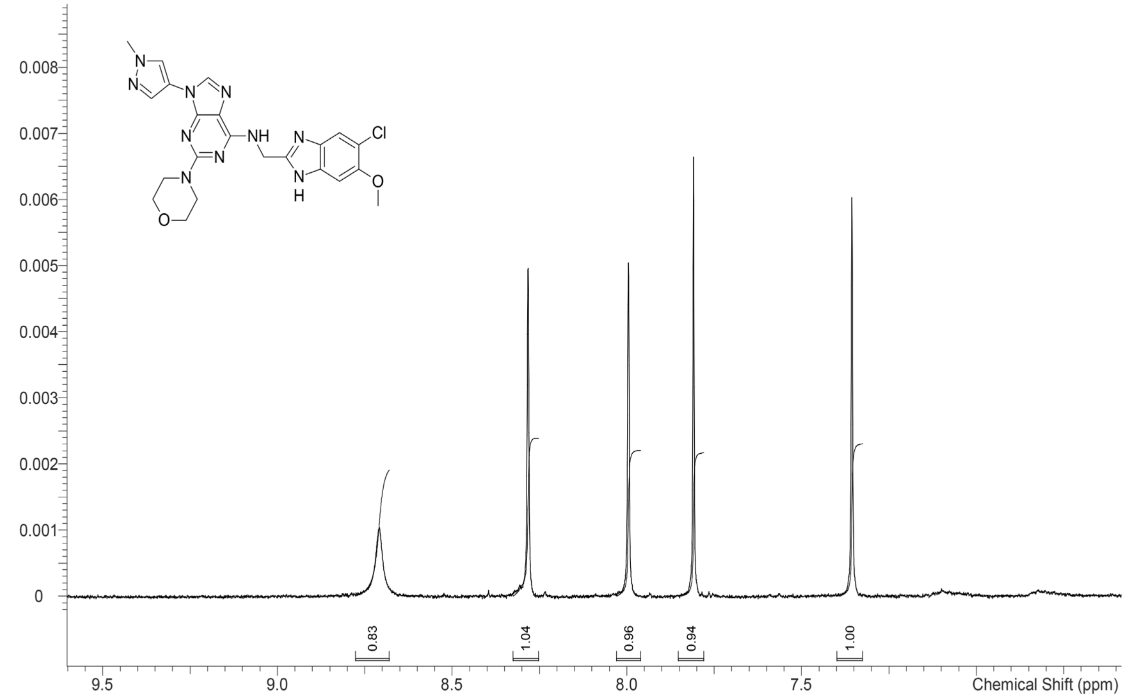

^{1}H NMR (400 MHz, CD_3OD) δ ppm 3.54 (br s, 8 H), 3.99 (s, 6 H), 5.19 (br s, 2 H), 7.36 (s, 1 H), 7.81 (s, 1 H), 8.00 (s, 1 H), 8.28 (s, 1 H), 8.71 (br s, 1 H).

Figure S13. 1H NMR spectrum of CU-0906
